# Functional epiphora: an under-reported entity

**DOI:** 10.1007/s10792-023-02668-4

**Published:** 2023-03-23

**Authors:** Eiman Usmani, Yinon Shapira, Dinesh Selva

**Affiliations:** 1 Discipline of Ophthalmology and Visual Science, University of Adelaide, Adelaide, South Australia Australia; 2 Department of Ophthalmology, Royal Adelaide Hospital and South Australian Institute of Ophthalmology, Adelaide, South Australia Australia

**Keywords:** Epiphora, Functional epiphora, Etiology, Nasolacrimal duct obstruction, Dacryocystography, Dacryoscintigraphy

## Abstract

**Purpose:**

To determine the etiology of epiphora in a tertiary Australian lacrimal clinic and highlight the high proportion of ‘functional’ cases.

**Methods:**

Single-center retrospective review: Records of adult patients presenting to a tertiary lacrimal clinic from January 2011 to February 2021 with epiphora were reviewed. Patients underwent testing with syringing/probing and lacrimal imaging to reach a diagnosis of functional epiphora. Functional epiphora was diagnosed based on the exclusion of alternate causes of epiphora on clinical examination, patent lacrimal syringing, normal dacryocystography, and delay on dacryoscintigraphy.

**Results:**

Five hundred and seventy-six symptomatic eyes of 372 adult patients (mean 66.2 ± 15.5 years, 63.4% females) with epiphora were evaluated for causes. Post-sac obstruction (stenosis/complete obstruction) and functional epiphora (non-anatomical delay) were the most common causes of presentations to the lacrimal clinic (26% each). Functional epiphora with post-sac delay was substantially more common than functional epiphora with pre-sac delay (89% vs. 11% of functional epiphora cases). In 16% of the cases, no cause for the epiphora was found while more than one cause (multifactorial) was present 11% of the time.

**Conclusion:**

Functional epiphora was found to be as common as a nasolacrimal anatomical obstruction when lacrimal imaging is utilized.

## Introduction

Studies investigating the etiology of epiphora have demonstrated post-sac drainage impairment (10–41%) [1–4], reflex tearing (29–52%) [1–5], and ‘multifactorial’ epiphora (19–29%) [4–7] to be the most common causes of presentations to their lacrimal clinics.

Post-sac lacrimal drainage impairment causes (nasolacrimal duct obstruction [NLDO], nasolacrimal duct stenosis [NLDS], or functional nasolacrimal duct delay [FNLDD]) are usually not differentiated and grouped under the same category [2–4, 7, 8] (e.g., “lacrimal system block” [3] or “lower lacrimal obstruction” [4]). Furthermore, there is a lack of standardization in diagnosing non-anatomical functional drainage impairment. In our practice, all suspected cases of impaired drainage undergo testing with dacryocystography (DCG) and dacryoscintigraphy (DSG), and based on the complementary findings of both imaging studies, ‘functional’ drainage impairment is determined.

There are only a few reports in the literature that highlight the high proportion of functional epiphora (i.e., non-anatomical impaired drainage) cases [9]. In a study by Wormald et al. [9], approximately 29% of the patients undergoing endoscopic dacryocystorhinostomy (DCR) for management of epiphora were diagnosed as having FNLDD on preoperative evaluation (utilizing DCG and DSG in the workup).

Thus, we aimed to retrospectively review the distribution of causes of epiphora in adult patients presenting to our lacrimal clinic to investigate the burden of functional epiphora in our population.

## Methods

Records of consecutive adult patients presenting to the Royal Adelaide Hospital lacrimal clinic with epiphora, from January 2011 to February 2021, were reviewed retrospectively. Cases that had previous lacrimal surgery were excluded. Only the symptomatic eyes were included in the analysis. The study received Institutional Review Board (IRB) approval and adhered to the tenets of the Declaration of Helsinki.

### Clinical evaluation

A thorough ocular history was undertaken of epiphora, including medication history, dacryocystitis, facial nerve palsy, ocular disease, sinus disease, allergies, frequency, and duration of symptoms.

This was followed by a comprehensive slit lamp clinical examination. Lids were assessed for the presence of eyelid margin disease (meibomian gland dysfunction, blepharitis, trichiasis), eyelid malposition or paralysis, dacryocystitis, mucoceles, or lagophthalmos. Causes of reflex tearing (evaporative dry eye disease using TBUT [< 10 s cutoff], allergic conjunctivitis, keratitis), punctal anomalies (size, location, and patency), and tear film height were evaluated.

Syringing/probing was performed using a 2 ml lacrimal cannula. The site of soft (indicating pre-sac obstructions) or hard stop and location and degree of reflux (%) were recorded. Patency on syringing was defined as less than 20% reflux. Finally, rigid nasal endoscopy was performed to assess potential nasal causes such as allergic rhinitis or rhinosinusitis.

### Imaging studies

Patients routinely underwent dacryocystography and dacryoscintigraphy as part of their assessment. These studies were performed by experienced radiologists and then assessed by expert oculoplastic specialists.

## DCG technique

For DCG, a drop of topical anesthetic (1% tetracaine hydrochloride) was instilled into the inferior conjunctival fornix of both eyes of patients in the supine position. A 27-gauge lacrimal cannula was then used to dilate the punctum. Following this, digital subtraction of the pre-contrast image from post-contrast images was achieved using baseline X-ray images and real-time imaging during injection of contrast (iopromide, Ultravist® 370; Bayer HealthCare Pharmaceuticals, Germany).

Patency through the system along with a duct diameter of less than 0.4 mm (width of a 27-gauge lacrimal cannula) on the X-ray image was defined as nasolacrimal duct stenosis [10]. No patency through the duct was termed an obstruction.

## DSG technique

For DSG, a gamma camera was used to create one-minute sequential images over 45 min. With the patient in the supine position, a 10 µl drop of technetium-99 m pertechnetate was placed into both eyes for the images. The participant was asked to clear their nasal passages if the tracer has not sufficiently progressed to reach the nasal cavity in any eye at the end of the serial scanning. This was followed by a lacrimal massage to both eyes. The imaging was then repeated and the location of the tracer at the lacrimal sac, lacrimal duct, or nasal cavity was recorded. The cutoff time-point to qualitatively determine normal versus pre-sac versus post-sac (NLD) delay based on end-tracer location was five minutes [10].

### Defining the cause of epiphora

The main cause for epiphora was designated based on the comprehensive evaluation detailed above. Multifactorial epiphora was diagnosed when more than one cause was determined to contribute. By definition, multifactorial epiphora did not include ‘functional epiphora’ as a potential constituent cause. Cases were designated as ‘unknown’ when the clinical examination was normal (no cause could be identified) and when at least one of the lacrimal imaging tests (DCG and/or DSG) was not available for analysis.

Functional epiphora was defined using Chan et al. [11] previously proposed stepwise algorithm:

1. Exclusion of alternate causes on clinical examination
2. Confirmed patency in syringing
3. Exclusion of nasolacrimal duct stenosis on DCG
4. Confirmed delay on DSG and location (i.e., functional epiphora with pre or post-sac delay)

Table 1 summarizes the definitions used in this study for the different types of post-sac lacrimal drainage impairment.

**Table 1:** Definitions of post-sac lacrimal drainage impairment based on findings of syringing, DCG and DSG

	*Syringing*	*DCG*	*DSG*
Nasolacrimal duct obstruction	Obstruction	Obstruction	Delay
Nasolacrimal duct stenosis	Patent	Stenosis	Delay
Functional nasolacrimal duct delay	Patent	Normal	Delay

^*^Dacryocystography (DCG)

^*^Dacryoscintigraphy (DSG)

## Results

**Fig. 1:**
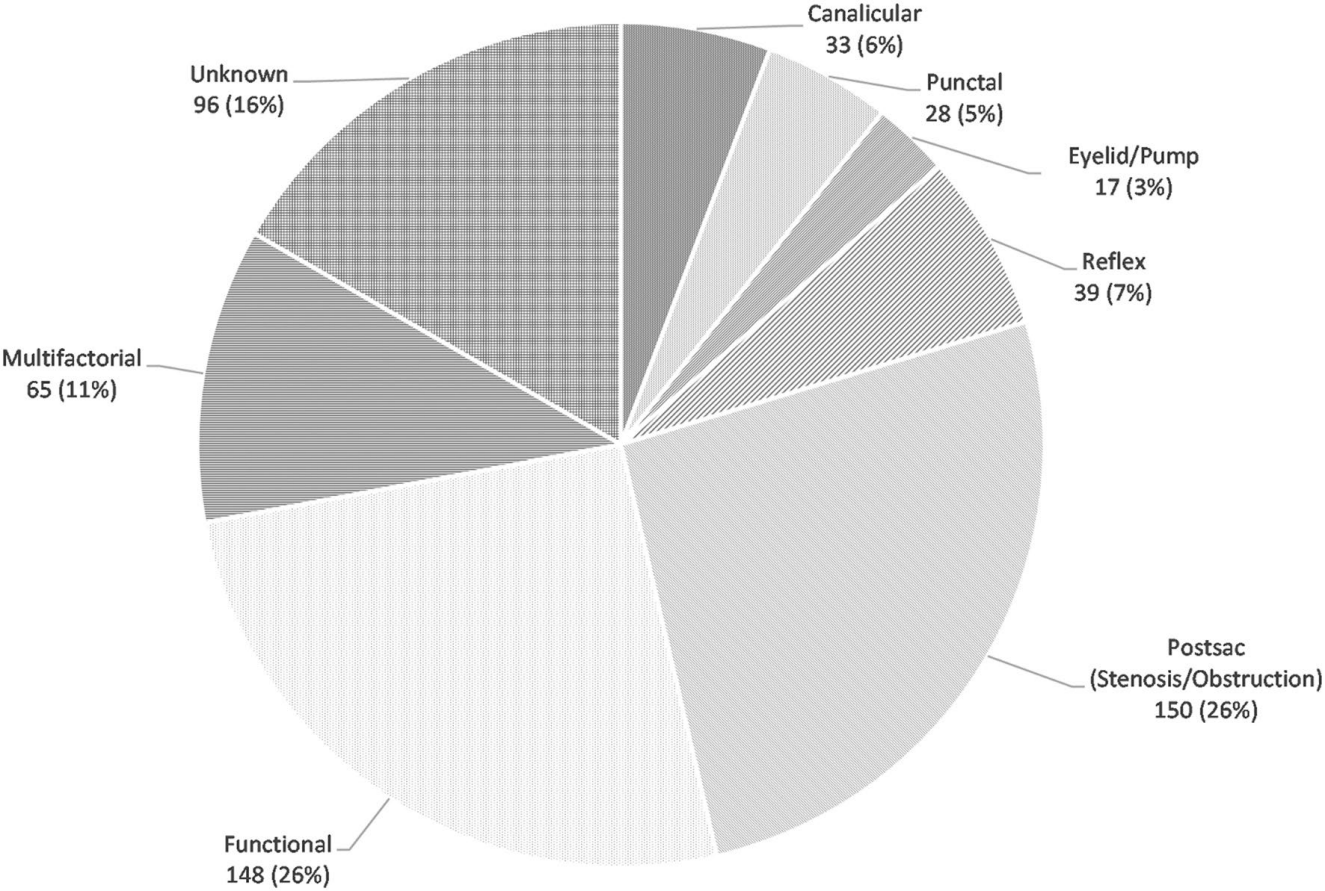
Distribution of all causes of epiphora among adult patients presenting to a tertiary lacrimal clinic. *Canalicular:* Stenosis or obstruction. *Punctal:* Malposition, stenosis, occlusion (e.g., secondary to conjunctivochalasis), *Eyelid/pump:* Eyelid malposition, laxity, facial nerve palsy, *Reflex*: Tear breakup time (TBUT) < 10 s, Blepharitis/meibomian gland dysfunction, Trichiasis, Keratitis, Allergic conjunctivitis, *Post-sac*: NLD stenosis, NLD Obstruction, *Functional:* Exclusion of other identifiable causes. Patent lacrimal syringing, normal DCG, and delay in DSG, *Multifactorial:* More than one cause found (excluding functional), *Unknown*—Normal clinical examination and at least one of the lacrimal imaging tests unavailable/uninterpretable

Five hundred and seventy-six symptomatic eyes of 372 patients presenting to our clinic from January 2011 to February 2021 with epiphora were evaluated for causes. The mean age of the patients was 66.2 ± 15.5 years (range 18–96 years) and 63.4% were females. See Fig. 1 for the distribution of the causes of epiphora.

Post-sac obstructions (stenosis/complete obstruction) were the most common etiology, accounting for 26% (150/576) of the cases, followed closely by functional epiphora (26%, 148/576). Of the 150 cases of post-sac obstructions, 76 (51%) of the lacrimal systems were diagnosed as nasolacrimal duct obstruction, and nasolacrimal duct stenosis was present in 74 (49%) of the cases. Post-sac delay made up 132/148 (89%) of the functional epiphora cases, whereas 16 (11%) of the functional epiphora cases were found to have a pre-sac delay.

In 16% (96/576) of the cohort, no cause for epiphora could be determined. More than one cause (multifactorial) was present 11% (65/576) of the time. Other causes included reflex epiphora (7%, 39/576), canalicular obstruction (6%, 33/576), punctal causes (5%, 28/576), and eyelid causes (3%, 17/576; malposition, laxity, or paralysis [pump failure]).

## Discussion

In the current study, the proportion of epiphora patients with functional epiphora and anatomical post-sac obstruction diagnosed based on clinical examination and lacrimal imaging were almost the same (26% of all causes). Our study demonstrates that, once routine imaging was utilized, functional epiphora (particularly FNLDD) was one of the most common causes of presentations to a tertiary lacrimal clinic.

The percentage of cases of post-sac anatomical obstruction/stenosis found in our clinic (26%) was slightly lower in comparison to numbers previously reported (up to ~ 40% [3]). The grouping of all causes of impaired lacrimal drainage may explain the overall higher frequencies reported in these studies [2–4]. Moreover, functional cases may be underrepresented in the literature (incidence reported ~ 1–7%) [1, 2, 5] as many are combined within the post-sac drainage impairment causes.

A few studies that attempt to differentiate cases of “patent but dysfunctional lacrimal systems,” diagnose these cases based on patency demonstrated either by syringing and/or using the jones test (once other causes have been excluded) [11]. However, clinical assessment alone cannot reliably differentiate between stenosis and FNLDD. Therefore, they probably include partial obstruction (stenosis) and non-anatomical functional delay in the same cohort [9, 11, 12].

Lacrimal syringing is a commonly used clinical test in the assessment of lacrimal drainage [13]. A study by Williams et al. [2] reported an incidence of 7% functional epiphora using “normal probing and irrigation” as the defining criterion. However, “lacrimal drainage system block” is used interchangeably with “nasolacrimal duct obstruction,” with a reported incidence of 33% [2]. In another study “partial or complete nasolacrimal duct obstruction” was described in 31.8% of cases using syringing [4]. Mainville [3] and Nemet et al. [6] utilized a combination of syringing and Jones testing to determine patency of the lacrimal system, with subsequent 40.7% of “lower system blockage” and 29% “nasolacrimal/canalicular obstruction,” respectively, reported. Nonetheless, our recent study showed that syringing as a sole criterion may not be reliable in differentiating between NLDS and FNLDD [14].

The Jones test is less commonly utilized [13], but it is considered by many as the “gold standard” for diagnosing a partial obstruction of the nasolacrimal duct and has been classically regarded to be the diagnostic test of choice for ‘functional obstruction’ [12, 15]. However, we are unaware of supportive evidence for the latter, for example by correlation with surgical findings (e.g., dacryoendoscopy or probing) and/or imaging [16]. Similarly, due to the lack of evidence, it could be argued that a combination of DCG and DSG does not provide a ‘gold standard’ method for determining the specific type of nasolacrimal duct dysfunction [10, 16]. Regardless, the complementary results of DCG and DSG may increase the sensitivity for diagnosing outflow impairment up to 98% [17]. Therefore, we believe it may provide a good preoperative adjunct for differentiating the cause of the dysfunction.

Perhaps clinicians do not meticulously attempt to differentiate the specific cause of nasolacrimal duct impairment as many will proceed to DCR regardless, if drainage impairment is suspected. However, intervention outcomes may vary between complete NLDO, NLDS, and FNLDD. For example, previous reports that did not distinguish between the non-anatomical functional block and nasolacrimal duct stenosis cases methodically reported different success rates (50–94%) of DCR in this group of patients [12, 18–22].

Some clinicians believe that lacrimal intubation is the treatment of choice for FNLDD. However, we have recently shown that endoscopic DCR was more successful than lacrimal intubation in resolving epiphora (65.2% vs. 34.1%) among patients with FNLDD [23]. Similarly, we found that DCR surgery may be more beneficial in patients with complete NLD obstruction as compared to stenosis (91% vs. 70%) [24]. These results suggest that clear diagnostic criteria may therefore be necessary for differentiation as this may have implications on the success of the surgery and is therefore relevant for counselling patients before surgery.

Finally, we acknowledge that variation in the prevalence of various nasolacrimal drainage pathologies may be due to geographical differences [25]. In a study by Bukhari et al. [1] conducted in Saudi Arabia, nasolacrimal duct obstruction was found in only 10.1% of the cases. Whereas pre-sac causes were only found in 11% of our cases, they were the most common etiology in their clinic (~ 45%) [1]. Similarly, ‘reflex tearing’ as the sole cause formed a small proportion of our cohort (7%), which is in contrast with some other populations where it was a more common finding (29–52%) [1–5].

A high incidence of pump dysfunction or reflex epiphora cases may have been expected given the older average age of our cohort. However, referrals to a tertiary lacrimal clinic may have led to exclusion of less complex causes as they may be well managed in the community setting. This may have caused these cases to be underrepresented in our study. Furthermore, the high proportion of functional epiphora may also represent a higher referral rate of such cases to a tertiary lacrimal clinic. Therefore, our results may not be generalizable to all populations or settings.

The strengths of the current study include a large cohort of over a decade combined with the use of a comprehensive clinical examination and imaging, all performed by experienced oculoplastic surgeons. This allowed differentiation between the specific causes of nasolacrimal duct drainage impairment using clear diagnostic criteria. However, the limitations of the current report should be acknowledged. In the current study, the diagnosis was determined based on initial assessment, and we do not present intraoperative findings or postoperative outcomes which is a limitation stemming from the retrospective design. In addition, clinical testing over the years may introduce inter-tester variations, even if standards were attempted to be maintained over time.

In conclusion, a high proportion of functional NLD delay cases were diagnosed once DCG and DSG were utilized in the evaluation of epiphora. FNLDD may have been combined with other causes of lacrimal drainage impairment and is hence underrepresented in the previous reports. It remains to be elucidated whether clearly defining the specific cause of drainage impairment via the use of lacrimal imaging-based criteria would affect the choice of intervention and/or its outcomes.
